# Use and Cost-Effectiveness of a Telehealth Service at a Centralized COVID-19 Quarantine Center in Taiwan: Cohort Study

**DOI:** 10.2196/22703

**Published:** 2020-12-11

**Authors:** Yung-Feng Yen, Yi-Fan Tsai, Vincent Yi-Fong Su, Shang-Yih Chan, Wen-Ruey Yu, Hsuan Ho, Chun-Mei Hou, Chu-Chieh Chen, Lin-Chung Woung, Sheng-Jean Huang

**Affiliations:** 1 Section of Infectious Diseases, Taipei City Hospital, Yangming Branch Taipei City Taiwan; 2 Department of Health Care Management National Taipei University of Nursing and Health Sciences Taipei Taiwan; 3 Institute of Public Health, National Yang-Ming University Taipei Taiwan; 4 University of Taipei Taipei Taiwan; 5 Department of Nursing Taipei City Hospital Yangming Branch Taipei Taiwan; 6 Department of Internal Medicine Taipei City Hospital Yangming Branch Taipei Taiwan; 7 School of Medicine, National Yang-Ming University Taipei Taiwan; 8 Department of Chest Medicine Taipei Veterans General Hospital Taipei Taiwan; 9 Department of Ophthalmology Taipei City Hospital Taipei Taiwan; 10 Department of Neurosurgery Taipei City Hospital Taipei Taiwan; 11 Department of Surgery, Medical College, National Taiwan University Hospital Taipei Taiwan

**Keywords:** COVID-19, international travelers, quarantine, telehealth, cost-effectiveness, cohort, monitoring, telemedicine

## Abstract

**Background:**

Telehealth is a recommended method for monitoring the progression of nonsevere infections in patients with COVID-19. However, telehealth has not been widely implemented to monitor SARS-CoV-2 infection in quarantined individuals. Moreover, studies on the cost-effectiveness of quarantine measures during the COVID-19 pandemic are scarce.

**Objective:**

In this cohort study, we aimed to use telehealth to monitor COVID-19 infections in 217 quarantined Taiwanese travelers and to analyze the cost-effectiveness of the quarantine program.

**Methods:**

Travelers were quarantined for 14 days at the Taiwan Yangmingshan quarantine center and monitored until they were discharged. The travelers’ clinical symptoms were evaluated twice daily. A multidisciplinary medical team used the telehealth system to provide timely assistance for ill travelers. The cost of the mandatory quarantine was calculated according to data from the Ministry of Health and Welfare of Taiwan.

**Results:**

All 217 quarantined travelers tested negative for SARS-CoV-2 upon admission to the quarantine center. During the quarantine, 28/217 travelers (12.9%) became ill and were evaluated via telehealth. Three travelers with fever were hospitalized after telehealth assessment, and subsequent tests for COVID-19 were negative for all three patients. The total cost incurred during the quarantine was US $193,938, which equated to US $894 per individual.

**Conclusions:**

Telehealth is an effective instrument for monitoring COVID-19 infection in quarantined travelers and could help provide timely disease management for people who are ill. It is imperative to screen and quarantine international travelers for SARS-CoV-2 infection to reduce the nationwide spread of COVID-19.

## Introduction

COVID-19 is caused by SARS-CoV-2 and was first detected in Wuhan, China, in December 2019. This disease caused a rapidly accelerating global pandemic in 2020 [1]; as of November 13, 2020, more than 51.8 million individuals were infected with COVID-19 globally, with the official death toll reaching 1.3 million [2].

As the COVID-19 outbreak emerged, the Taiwanese government implemented several strategies to prevent the nationwide spread of COVID-19, including border controls, proactive screening measures, and quarantine procedures [3,4]. According to the Taiwan Communicable Disease Control Act [5], as of January 2020, all international travelers who visited regions with a declared COVID-19 outbreak were required to complete a 14-day mandatory quarantine. By November 13, 2020, 597 laboratory-confirmed COVID-19 cases were reported to the Taiwan Centers for Disease Control, including 505 imported cases (84.6%) [6]. The national mortality rate among laboratory-confirmed COVID-19 cases was 1.2% [6].

Currently, no effective pharmacological interventions or vaccines are available to treat or prevent COVID-19 [7,8]. Therefore, nonpharmacological public health measures such as quarantine, isolation, social distancing, and community containment are the only effective ways to prevent infection and control the COVID-19 outbreak [9]. Quarantine is the most effective tool for controlling the COVID-19 outbreak [9]; it refers to the restriction of asymptomatic healthy people who have had contact with confirmed or suspected COVID-19 cases. Quarantine can be voluntary or mandatory, and it can be applied at an individual or group level. A recent Cochrane review [10] showed that the quarantine strategy could significantly reduce the number of people infected with SARS-CoV-2 and decrease the number of COVID-19–related deaths.

During quarantine, all individuals should be monitored for the onset of symptoms; otherwise, there is risk of delays in the detection and prompt management of the virus. Telehealth through a line of communication is an efficient way to monitor quarantined individuals and can assist in providing timely care for people who require it [11]. Although telehealth has been recommended to screen patients with COVID-19 in emergency departments [12] and provide care for nonsevere COVID-19 cases [13], it has not been widely implemented in the monitoring of COVID-19 infection in quarantined individuals.

Quarantine strategies have been used in many countries in an attempt to curb the ongoing COVID-19 pandemic. However, the cost-effectiveness of these strategies has not been extensively studied [10]. A current report [14] indicates that the quarantine strategy is efficient in curbing the spread of COVID-19, and the cost of a quarantine strategy is lower than that of lockdown of workplaces.

This cohort study reports on a model used for screening and quarantining Taiwanese travelers following an evacuation flight from Hubei, China. In this model, telehealth was used to monitor COVID-19 infection among travelers at a centralized quarantine center, and the cost-effectiveness of the 14-day mandatory quarantine strategy was analyzed.

## Methods

### Background Information and Study Subjects

In late January 2020, the Taiwanese government established a centralized quarantine center to screen and monitor COVID-19 infections in international travelers who had visited countries with a declared COVID-19 outbreak [5]. On March 30, 2020, the government of Taiwan arranged a special charter flight to evacuate Taiwanese travelers who had become stranded in Hubei, China, and return them to Taiwan. Upon arrival in Taiwan, the travelers proceeded to one of the largest national centralized quarantine centers, Taipei Yangmingshan, for the mandatory 14-day quarantine period. The Yangmingshan quarantine center included six quarantined wards on three floors.

This cohort study included all the passengers of the charter flight, who were subsequently followed up until their discharge from the quarantine center or until April 15. This study was approved by the Institutional Review Board of Taipei City Hospital (no. TCHIRB-10904014-E).

### Infection Control Strategies

The body temperature of the Taiwanese travelers was checked at the international airport in China, and individuals with fever were not allowed to board the charter flight. All travelers were required to disinfect their hands with alcohol-based hand sanitizer at the boarding port and to wear personal protective equipment (PPE) provided by the Taiwanese government, which included a face mask and medical gown. Meals were not provided during the flight to avoid cross-infection between passengers.

Upon arrival at the international airport in Taiwan, National Defense chemical troops used a 1:10 diluted sodium hypochlorite solution (5000 ppm) to disinfect the travelers’ luggage. The travelers then proceeded to the Yangmingshan quarantine center for the 14-day quarantine period. They were required to record their body temperature twice daily with a thermometer that was provided to them. Additionally, daily meals were delivered to the individuals’ rooms, and janitors used a 1:10 diluted sodium hypochlorite solution to clean the quarantine center once daily.

### COVID-19 Screening and Monitoring

During admission to the Yangmingshan centralized quarantine center, the travelers were screened for COVID-19 using a real-time reverse transcriptase–polymerase chain reaction (RT-PCR) test. If an individual tested positive for COVID-19, they were transferred to a hospital for medical isolation and treatment.

Travelers’ clinical symptoms were recorded twice daily by their primary care nurses via telephonic communication. If an individual developed fever or became ill, a multidisciplinary team (MDT) consisting of 15 medical workers used telehealth to assess the traveler’s condition.

### Telehealth System

The telehealth system at the Yangmingshan quarantine center was developed based on a popular social media app called LINE, which is a freeware app operated by the NAVER Corporation in South Korea. The LINE-based telehealth system established two-way communication between the MDT and 217 quarantined travelers. Through a unique ID, the quarantined individuals were invited to join an official LINE group in which the quarantined travelers could report their symptoms to the MDT members. When the MDT members received a message regarding a quarantined traveler’s symptoms, the MDT members initiated a one-to-one video call through LINE to clinically evaluate the patient. The MDT members collaboratively discussed the traveler’s condition during the telehealth session and provided appropriate management and treatment interventions for the traveler. If a quarantined traveler presented with fever or shortness of breath, they were transferred to the hospital for further treatment.

### Personnel and Cost at Yangmingshan Quarantine Center

The personnel who were responsible for overseeing the travelers’ quarantine at the Yangmingshan quarantine center included the MDT, police officers, janitors, a logistics group, and administration staff. The MDT consisted of 3 physicians, 10 nurses, a pharmacist, and a psychologist. Three physicians, including a general practitioner, a pediatrician, and a cardiologist, gave treatment advice according to the traveler’s condition during the telehealth session. The 10 nurses were responsible for taking care of the travelers during 12-hour day and night shifts in the six quarantined wards. The nurses communicated daily with the quarantined travelers to evaluate their clinical condition and record their body temperature. The pharmacist delivered medications to ill quarantined travelers. Physicians and nurses wore PPE when examining the travelers in person.

The police officers were responsible for the security of the travelers at five sentry points and ensured that all travelers obeyed the COVID-19 quarantine rules and completed the 14-day mandatory quarantine order. In total, 18 police officers were divided into three groups, with one leader in each group. The three groups of police officers worked in 12-hour day and night shifts. Three janitors were responsible for environmental disinfection in the six quarantined wards, and one janitor was responsible for two quarantined wards on one floor. The logistics group, composed of 10 people, was responsible for meal preparation and ensuring that sufficient supplies were provided to the 217 travelers during the 14-day quarantine.

The cost of the quarantine for the travelers at the Yangmingshan quarantine center was covered by the disaster reserve of the national Ministry of Health and Welfare, which included payment of the MDT, nonmedical personnel, and telehealth services as well as the provision of PPE.

### Statistical Analysis

First, the participants’ demographic data were analyzed. Continuous data are presented as mean (SD), and two-sample *t* tests were used for comparisons between groups. Categorical data were analyzed with Pearson chi-square tests where appropriate. A *P* value <.05 was considered to indicate statistical significance.

A timeline infographic was used to display the progression of clinical symptoms in hospitalized travelers. The labor and overall costs of the quarantine were calculated according to the disaster reserve data from the Taiwan Ministry of Health and Welfare. All data management and analyses were performed using SPSS 19.0 (IBM Corporation).

## Results

### Participant Selection and Epidemiologic Features

**Figure 1:**
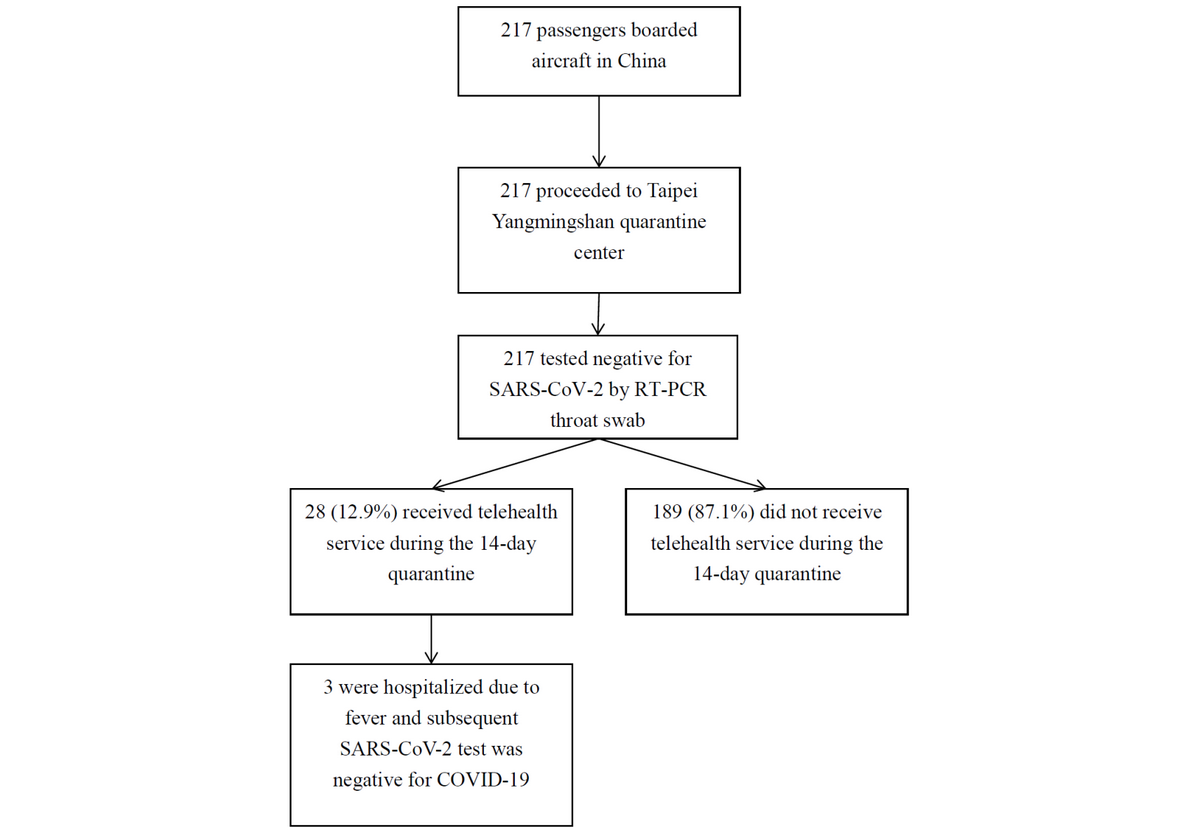
The process of follow-up in Taiwanese travelers quarantined at the Yangmingshan centralized quarantine center. RT-PCR: reverse transcriptase–polymerase chain reaction.

Our sample comprised 217 Taiwanese travelers who were stranded in Hubei, China, due to the COVID-19 pandemic and who returned to Taiwan on March 30, 2020 (Figure 1). The mean age of the sample was 30.0 years (SD 19.4), and 130 of the 217 subjects (59.9%) were female. All travelers tested negative for COVID-19 by an RT-PCR throat swab upon admission to the Taipei Yangmingshan quarantine center. During the 14-day quarantine, 28 of the 217 travelers (12.9%) underwent telehealth consultations due to illness.

### Characteristics of Quarantined Travelers With and Without Telehealth Evaluation

Table 1 shows the characteristics of quarantined travelers who were and were not evaluated with telehealth. There was no significant difference in the age or sex of the quarantined travelers who were and were not evaluated with telehealth. Three travelers had fever and underwent clinical assessment using telehealth during the 14-day quarantine period.

**Table 1:** Characteristics of Taiwanese travelers who were and were not evaluated with telehealth at a centralized quarantine center.

Characteristic		Total (N=217)		Evaluated with telehealth (n=28)		Not evaluated with telehealth (n=189)		*P* value	
Age (years), mean (SD)		30.0 (19.40)		33.0 (18.1)		29.6 (17.3)		0.33	
**Age (years), n (%)**									0.68
	<20		78 (35.9)		8 (28.6)		70 (37.0)		
	20-39		54 (24.9)		8 (28.6)		46 (24.3)		
	≥40		85 (39.2)		12 (42.8)		73 (38.7)		
**Sex, n (%)**									0.61
	Female		130 (59.9)		10 (35.7)		77 (40.7)		
	Male		87 (40.1)		18 (64.3)		112 (59.3)		
**Number of family members, n (%)**									<.001
	1		79 (36.4)		19 (67.9)		60 (31.7)		
	≥2		138 (63.6)		9 (32.1)		129 (68.3)		
**Fever during the 14-day quarantine, n (%)**									<.001
	No		214 (98.6)		25 (89.3)		189 (100)		
	Yes		3 (1.4)		3 (10.7)		0 (0)		
**Hospitalization, n (%)**									<.001
	No		214 (98.6)		25 (89.3)		189 (100)		
	Yes		3 (1.4)		3 (10.7)		0 (0)		

### Symptoms and Management in Quarantined Travelers Receiving Telehealth Care

The symptoms and management of quarantined travelers receiving telehealth care were recorded during the 14-day mandatory quarantine (Multimedia Appendix 1). The most common symptoms requiring telehealth consultations in quarantined travelers were fever (n=3), diarrhea (n=3), toothache (n=3), and skin rashes (n=3). Of the 217 travelers, 3 (1.4%) developed fever during the 14-day quarantine period and were hospitalized after a telehealth assessment.

Of the 28 quarantined travelers evaluated with telehealth, one traveler with diabetes presented with dizziness and weakness on the fifth day and was suspected to be hypoglycemic during the telehealth clinical evaluation. The patient’s blood glucose test indicated a level of 75 mg/dl when health care workers wearing PPE visited this individual.

### Clinical Features of Hospitalized Patients

**Figure 2:**
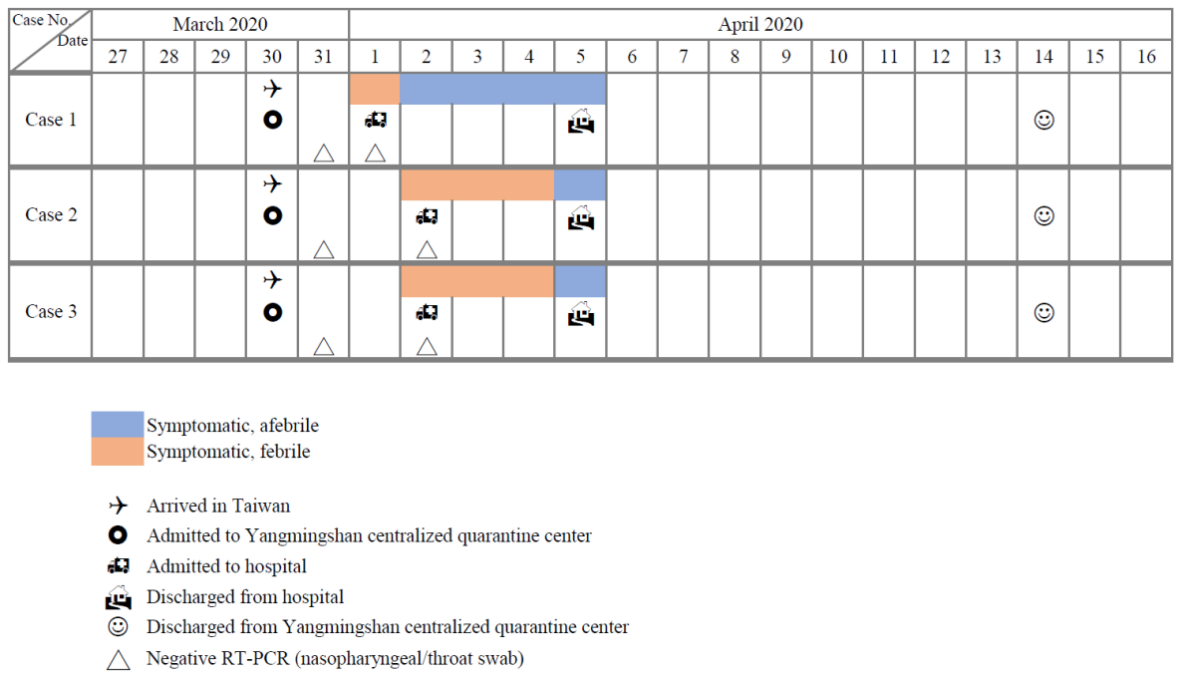
Time course of clinical symptoms in the three hospitalized travelers.

Figure 2 shows the progression of clinical symptoms in three hospitalized travelers. One traveler developed fever and illness 2 days after quarantine, and two additional travelers developed fever 3 days after being admitted to the centralized quarantine center. Chest x-rays of all three patients were negative for pneumonia. Moreover, a second round of SARS-CoV-2 testing by RT-PCR throat swab was negative for COVID-19 in all three individuals. They were subsequently discharged from the hospital on April 5 and returned to the Yangmingshan quarantine center to complete the mandatory quarantine.

### Cost Analysis of the 14-Day Mandatory Quarantine of Taiwanese Travelers

Table 2 shows the labor and overall cost incurred during the 14-day mandatory quarantine. The major personnel consisted of police officers (n=18), followed by the MDT (n=15). Moreover, the major expense was the labor cost of the MDT (US $72,334, 37.30%), followed by police officers (US $66,427, 34.25%) and the cost of PPE (US $16,016, 8.26%). The total cost incurred during the 14-day quarantine was US $193,938, which equated to US $894 per traveler.

**Table 2:** Cost analysis of the 14-day mandatory quarantine of 217 Taiwanese travelers.

Variable		Personnel, n	Cost (US $), amount (%)
**Personnel costs**			
	Multidisciplinary medical team	15	72,334 (37.30)
	Police officers	18	66,427 (34.25)
	Janitors	3	10,880 (5.61)
	Logistic group	10	8033 (4.14)
	Administration staff	1	894 (0.46)
**Nonpersonnel costs**			
	Telehealth equipment	N/A^a^	2838 (1.46)
	Personal protective equipment	N/A	16,016 (8.26)
	Disinfecting equipment	N/A	115 (0.06)
	Infectious waste disposal	N/A	1400 (0.72)
	Staff uniform disinfection	N/A	6667 (3.44)
	Meals and daily supplies for quarantined travelers	N/A	8334 (4.30)
Total		47	193,938 (100)

^a^N/A: not applicable.

## Discussion

### Principal Findings

This cohort study reports a model for the screening and quarantining of international travelers who visited countries with a declared COVID-19 outbreak. All travelers tested negative for COVID-19 during the 14-day mandatory quarantine period. Three travelers with fever were hospitalized after the telehealth assessment, and subsequent tests for COVID-19 were negative for all three patients. The total costs during the quarantine were US $193,938, which equated to US $894 per individual. Our study demonstrates that strict infectious control measures, proactive screening, and an MDT integrated with the use of telehealth contributed to the successful quarantine of international travelers while remaining cost-effective for containing the spread of COVID-19.

Quarantine is an effective strategy to control and prevent COVID-19 outbreaks [9]. However, monitoring individuals in quarantine is essential to provide prompt management and early detection of COVID-19 cases. By establishing a line of communication between health care workers and quarantined individuals, telehealth can provide timely assessment of quarantined individuals and fast-track the hospitalization of people who develop symptoms of COVID-19. Although telehealth has not been widely adopted in monitoring COVID-19 infections in quarantined individuals, one study in China [15] used a telehealth system to monitor 188 home-quarantined individuals; it was found that 74 individuals (39.4%) were infected with SARS-CoV-2. Moreover, 6 of the 74 confirmed COVID-19 cases (8.1%) were hospitalized after the telehealth assessment [15]. Our study used telehealth to monitor COVID-19 infections in 217 international travelers during the 14-day mandatory quarantine. Three travelers with fever were hospitalized after the telehealth assessment, and subsequent tests for COVID-19 were negative for all three patients. Because telehealth can assist in providing timely assessment and does not delay hospitalization of quarantined individuals, our study suggests that it is imperative to adopt telehealth to monitor COVID-19 infections in this population.

Our report is among the first to analyze the cost-effectiveness of an enforced quarantine program. We found that the total cost of a 14-day quarantine for 217 international travelers was US $193,938, equating to US $894 per traveler. Although none of the 217 Taiwanese travelers tested positive for SARS-CoV-2 during the 14-day mandatory quarantine period, this study highlights the importance of screening and immediately quarantining returning international travelers because of the high risk of COVID-19 in this population [16]. The quarantine strategy in our report is also corroborated by a recent study [14], in which it was reported that the quarantine strategy is efficient in curbing the COVID-19 outbreak, and its relative efficacy increases when supplemented with other measures designed to reduce disease transmission.

LINE-based telehealth can provide timely assessment and care for patients who require it. During the COVID-19 surge, LINE-based telehealth could provide care for nonsevere COVID-19 cases in hospitals [13], and health care workers can provide timely assessment and management of patients with COVID-19 through the telehealth system and reduce the transmission of SARS-CoV-2 in health care facilities [11].

### Limitations

The present study has two limitations. First, our study did not compare the cost-effectiveness of a 14-day quarantine for high-risk COVID-19 individuals. In this cohort study, none of the 217 Taiwanese travelers tested positive for SARS-CoV-2 during the 14-day mandatory quarantine period. As SARS-CoV-2 is highly contagious [17], it is imperative to screen and quarantine travelers who have visited countries with a declared COVID-19 outbreak. Second, the external validity of our findings may be a concern because all our patients were Taiwanese. The generalizability of our results to other non-Asian ethnic groups requires further verification. However, our findings suggest new avenues for future research.

### Conclusion

This prospective cohort study reports an enforced quarantine program integrated with a telehealth system to monitor COVID-19 infection in quarantined individuals. Telehealth not only provided timely management of quarantined individuals with illness but also reduced the risk of COVID-19 infection in health care workers. Our study suggests that it is imperative to screen and quarantine international returning travelers to reduce the nationwide spread of COVID-19.

## Appendix

Multimedia Appendix 1 Symptoms and telehealth management of 28 quarantined travelers.

## Abbreviations

PPE: personal protective equipment
RT-PCR: reverse transcriptase–polymerase chain reaction
MDT: multidisciplinary team
