# Supplementary material for: Use and Cost-Effectiveness of a Telehealth Service at a Centralized COVID-19 Quarantine Center in Taiwan: Cohort Study
Source: J Med Internet Res. 2020 Dec 11;22(12):e22703. doi: 10.2196/22703 (PMC7735809; doi:10.2196/22703)
Supplement: Multimedia Appendix 1 [file jmir_v22i12e22703_app1.docx]

| **Multimedia Appendix 1.** Symptoms and management in 28 quarantined travelers with telehealth. | | |
| --- | --- | --- |
| Symptoms/Management | n | % |
| Fever/Hospitalization | 3 | 10.71 |
| Diarrhea/Anti-diarrhea drugs | 3 | 10.71 |
| Toothache/Nonsteroidal anti-inflammatory drugs | 3 | 10.71 |
| Skin rashes/Anti-fungal and steroid agents | 3 | 10.71 |
| Dizziness/Finger-stick blood glucose test | 1 | 3.57 |
| Insomnia/Hypnotic drug | 2 | 7.14 |
| COVID-19-related stress/Caring and emotional support | 2 | 7.14 |
| Pregnancy-related sickness/Evaluation of pregnancy | 2 | 7.14 |
| Oral herpes/Acyclovir cream | 1 | 3.57 |
| Allergy/Antihistamine agent | 1 | 3.57 |
| Hypertension/Anti-hypertensive drugs | 1 | 3.57 |
| Blepharitis/Warm compress | 1 | 3.57 |
| Sprained right ankle/Nonsteroidal anti-inflammatory drugs | 1 | 3.57 |
| Falling accident/Clinical evaluation | 2 | 7.14 |
| Forehead lacerative wound/Wound care | 1 | 3.57 |
| Burn injury/Silver sulfadiazine cream treatment | 1 | 3.57 |
| COVID-19, coronavirus disease 2019. | | |
